# Supplementary material for: Paper-based electrochemical device for early detection of integrin αvβ6 expressing tumors
Source: Commun Chem. 2024 Mar 21;7:60. doi: 10.1038/s42004-024-01144-z (PMC10957923; doi:10.1038/s42004-024-01144-z)
Supplement: Supplementary file 2 — Supplementary Information File [file 42004_2024_1144_MOESM2_ESM.pdf]

Paper-based Electrochemical Device for Early Detection of Integrin αvβ6 Expressing Tumors

Stefano Cinti*§[a], Stefano Tomassi§[a], Chiara Ciardiello§[b], Rossella Migliorino [b], Marinella Pirozzi[c], Alessandra Leone [b], Elena Di Gennaro [b], Virginia Campani[a], Giuseppe De Rosa[a], Vincenzo Maria D'Amore[a], Salvatore Di Maro[d], Greta Donati[a], Sima Singh[a], Ada Raucci[a], Francesco Saverio Di Leva[a], Horst Kessler[e], Alfredo Budillon[f], Luciana Marinelli*[a]

[a] Dipartimento di Farmacia, Università degli Studi di Napoli "Federico II", Via D. Montesano 49, 80131, Naples, Italy
[b] Experimental Pharmacology Unit, Istituto Nazionale Tumori –IRCCS– Fondazione G. Pascale, 80131, Naples, Italy
[c] Second Unit, Institute of Experimental Endocrinology and Oncology "G. Salvatore" (IEOS), National Research Council (CNR), Naples, Italy.
[d] DiSTABiF, Università degli Studi della Campania Luigi Vanvitelli, Via Vivaldi 43, 81100 Caserta (Italy)
[e] Institute for Advance Study, Department of Chemistry, Technical University of Munich, Garching, Germany.
[f] Scientific Director, Istituto Nazionale Tumori –IRCCS– Fondazione G. Pascale, 80131, Naples, Italy

§ These authors equally contributed to this work.

Table of Contents

1. **Scheme S1.** Synthetic scheme of cyclic peptides **2-5** **2**

2. **Table S1**. Analytical chromatographic procedure **3**

3. **Figure S1** Analytical data for compounds **2-5.** **4-7**

4. **Figure S2** Western Blot on cells and Immunoelectron microscopy negative control **8**

5. **Figure S3.** Uncropped blot for Figures 5c and 5d **9**

6. **Figure S4.** Cyclic voltammograms **10**

7. **Table S2**: Comparison with other biosensors/biosensing approaches **11**

Scheme S1. Detailed solid phase synthetic scheme for compounds **2-5**.

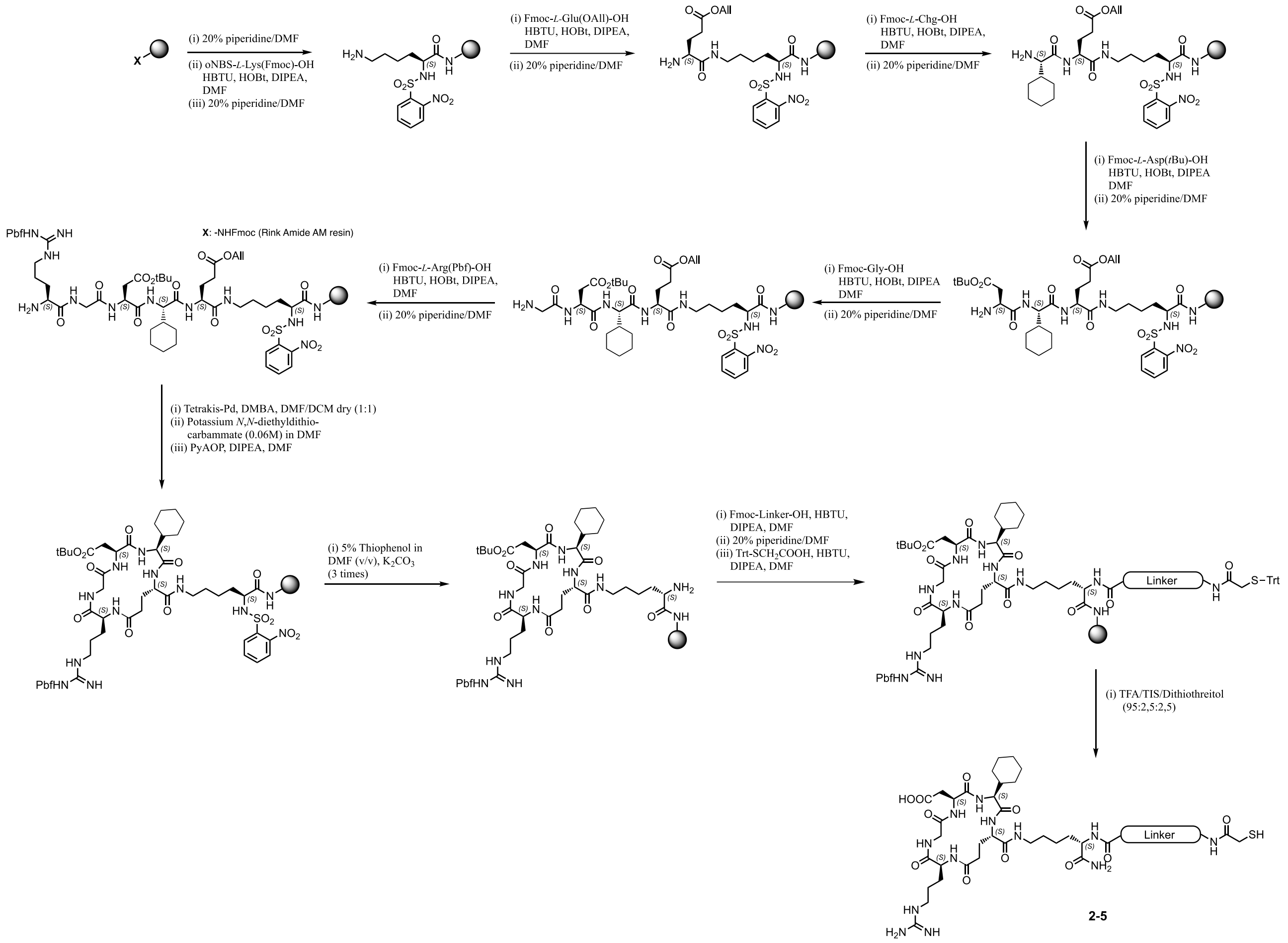

Table S1. Analytical chromatographic procedure.

Time interval		% of AcCN in water
0 min	5 min	10
5 min	25 min	90
25 min	30 min	90
30 min	33 min	10
33 min	35 min	10

Figure S1. Analytical data of probes 2-5

Probe 2

25 mg, crude yield: 50 %, purity: ≥ 95%, t_R 12.11 min, (analytical HPLC, 10 to 90% acetonitrile (0.1% TFA) in water (0.1% TFA) over 20 min, flow rate of 1.0 mL/min); LRMS (ESI-MS): calculated: 883.45 for $C_{37}H_{63}N_{12}O_{11}S$ $[M+H]^{+}$, found: 883.62. Calculated: 442.23 for $C_{37}H_{64}N_{12}O_{11}S$ $[M+2H]^{2+}/2$, found: 442.52.

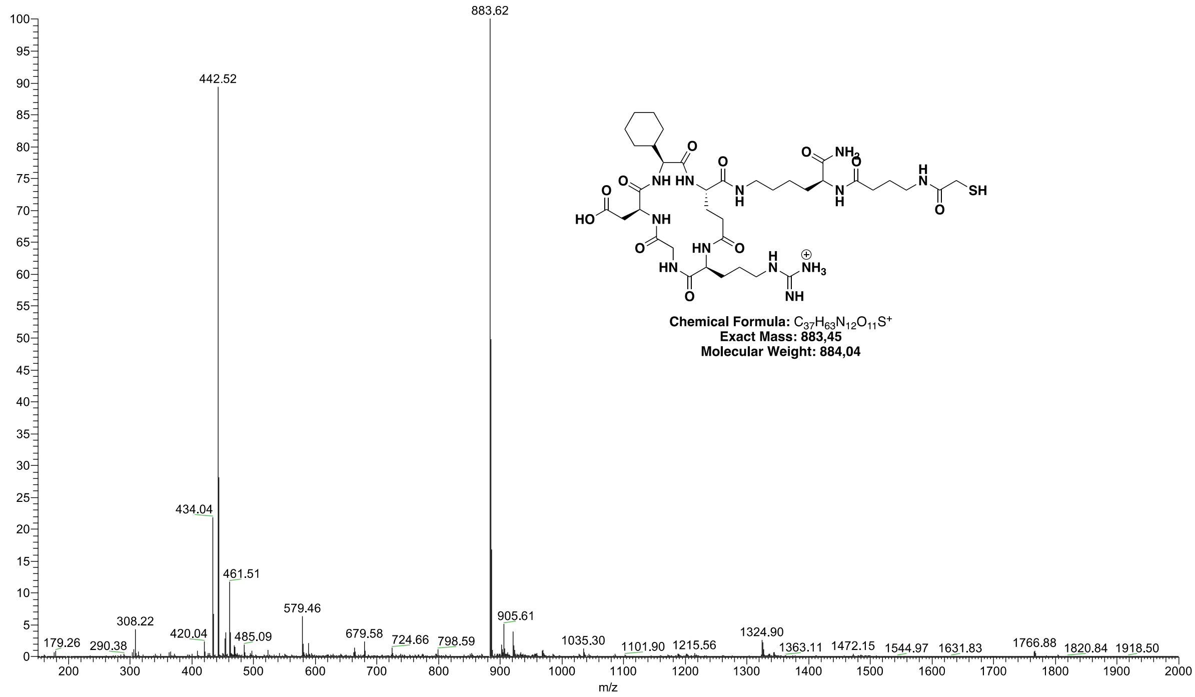

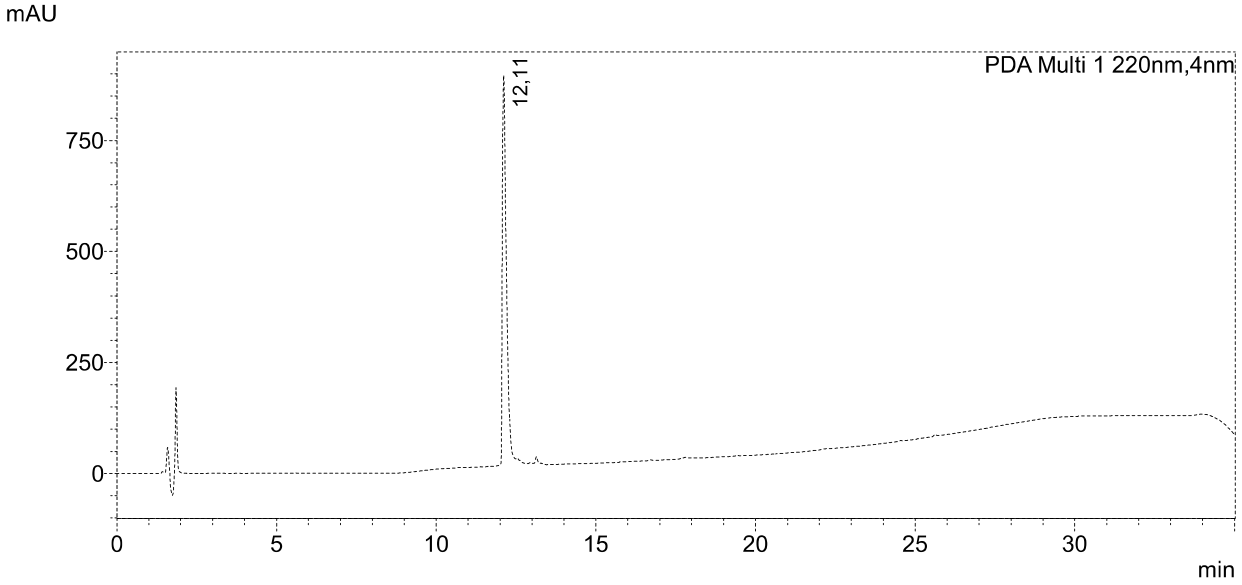

Probe 3

28 mg, crude yield: 53 %, purity: ≥ 95%, t_R 12.16 min, (analytical HPLC, 10 to 90% acetonitrile (0.1% TFA) in water (0.1% TFA) over 20 min, flow rate of 1.0 mL/min); LRMS (ESI-MS): calculated: 943.47 for $C_{39}H_{67}N_{12}O_{13}S$ $[M+H]^+$, found: 943.67. Calculated: 472.24 for $C_{39}H_{68}N_{12}O_{13}S$ $[M+2H]^{2+}/2$, found: 472.57.

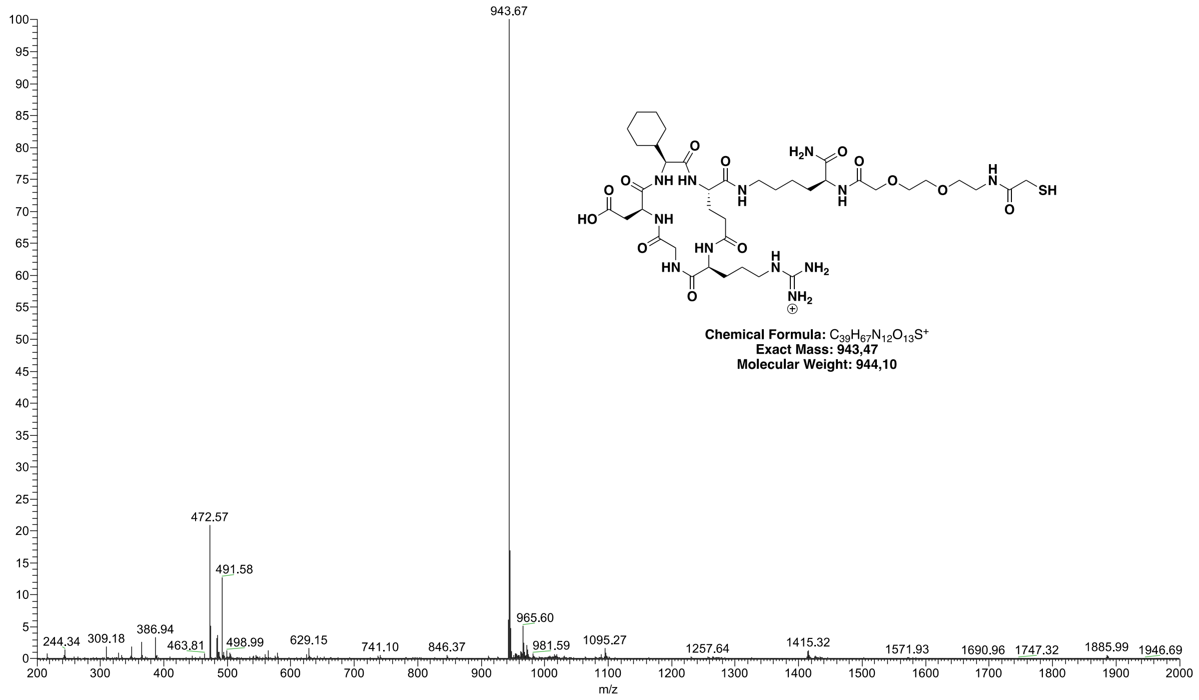

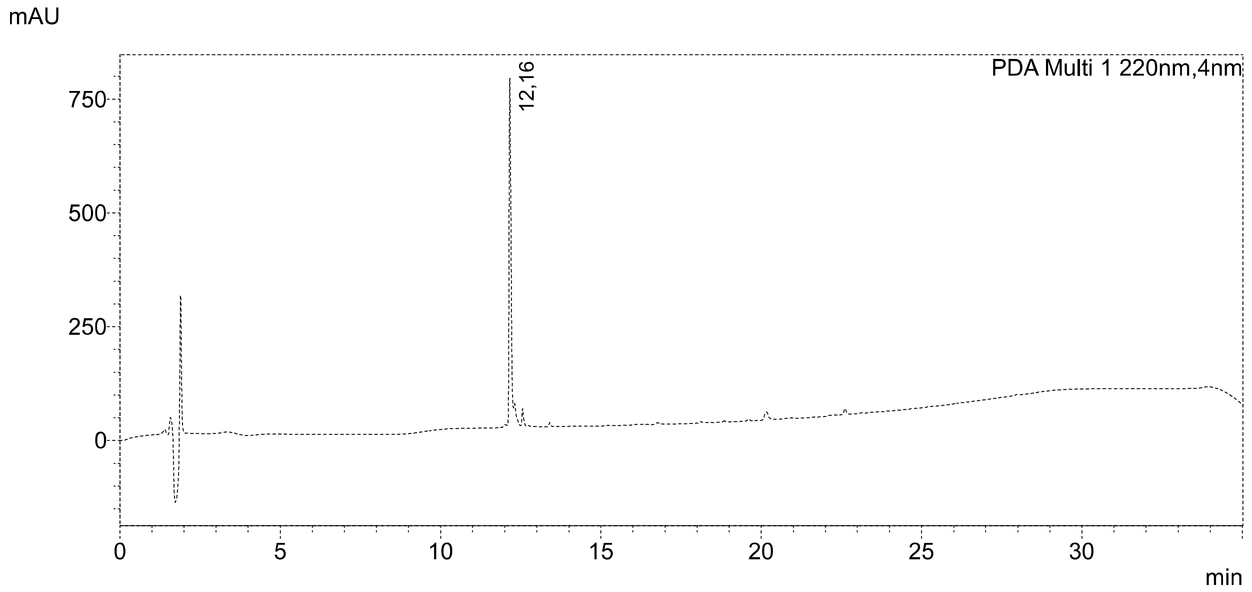

Probe 4

34 mg, crude yield: 60 %, purity: ≥ 90%, t_R 12.51 min, (analytical HPLC, 10 to 90% acetonitrile (0.1% TFA) in water (0.1% TFA) over 20 min, flow rate of 1.0 mL/min); LRMS (ESI-MS): calculated: 1028.52 for $C_{43}H_{74}N_{13}O_{14}S$ $[M+H]^+$, found: 1028.81. Calculated: 514.76 for $C_{43}H_{75}N_{13}O_{14}S$ $[M+2H]^{2+}/2$, found: 515.12.

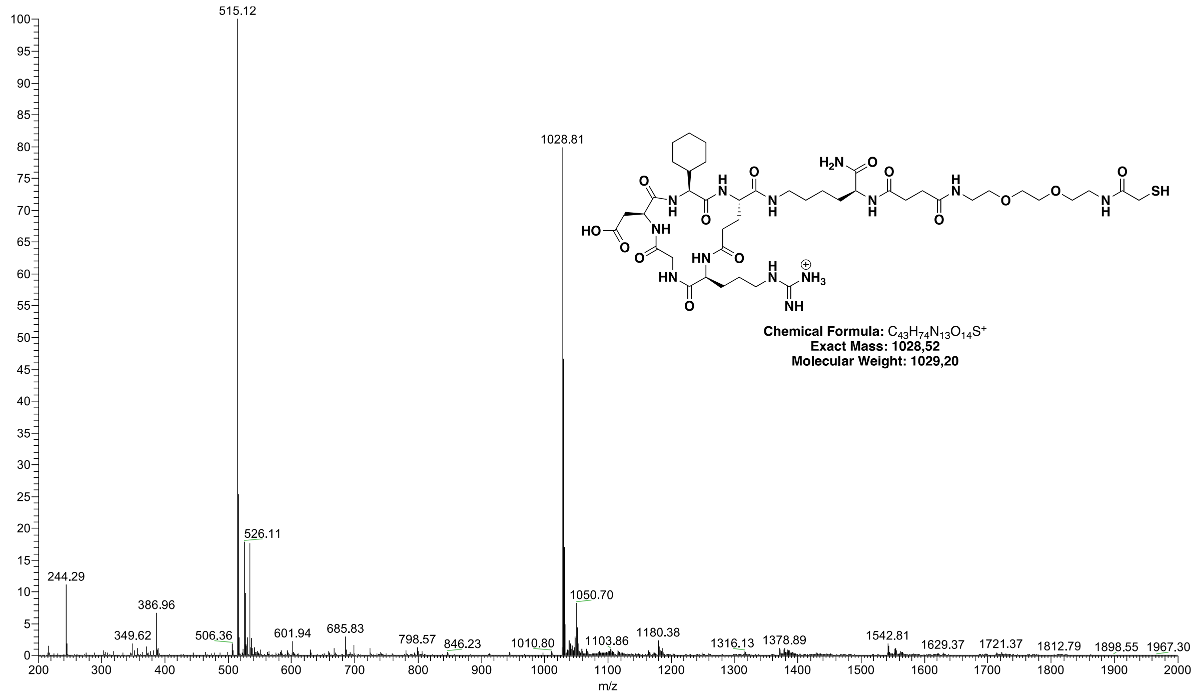

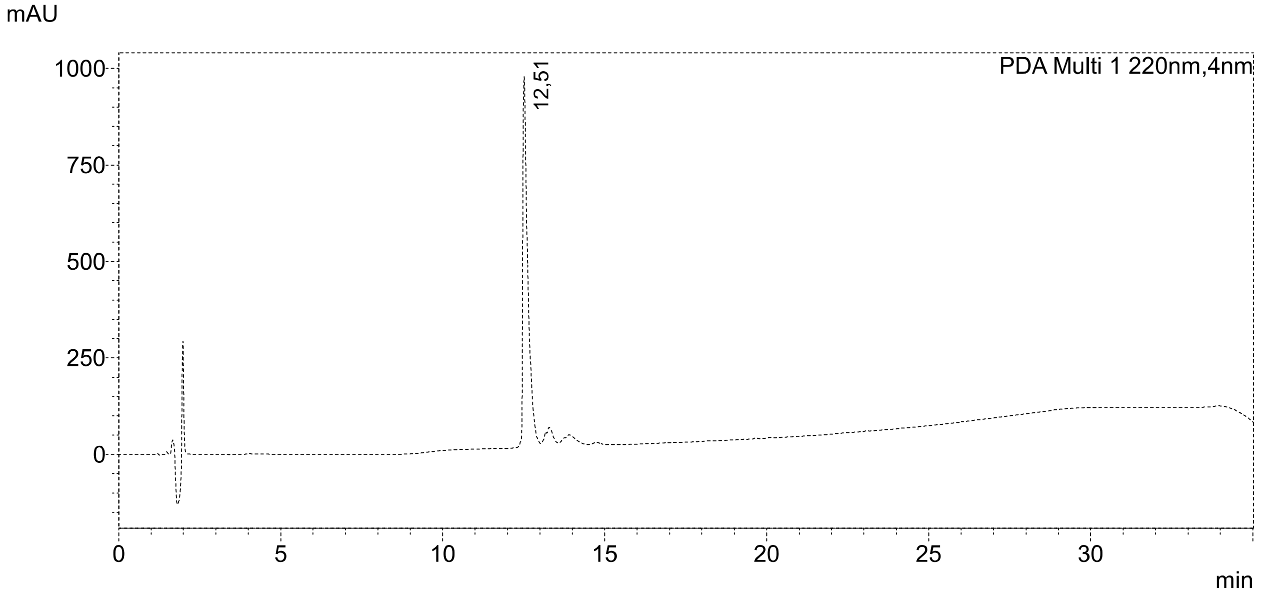

Probe 5

36 mg, crude yield: 58 %, purity: ≥ 90%, t_R 13.16 min, (analytical HPLC, 10 to 90% acetonitrile (0.1% TFA) in water (0.1% TFA) over 20 min, flow rate of 1.0 mL/min); LRMS (ESI-MS): calculated: 1116.57 for $C_{47}H_{82}N_{13}O_{16}S$ $[M+H]^+$, found: 1116.76. Calculated: 558.79 for $C_{47}H_{83}N_{13}O_{16}S$ $[M+2H]^{2+}/2$, found: 559.19.

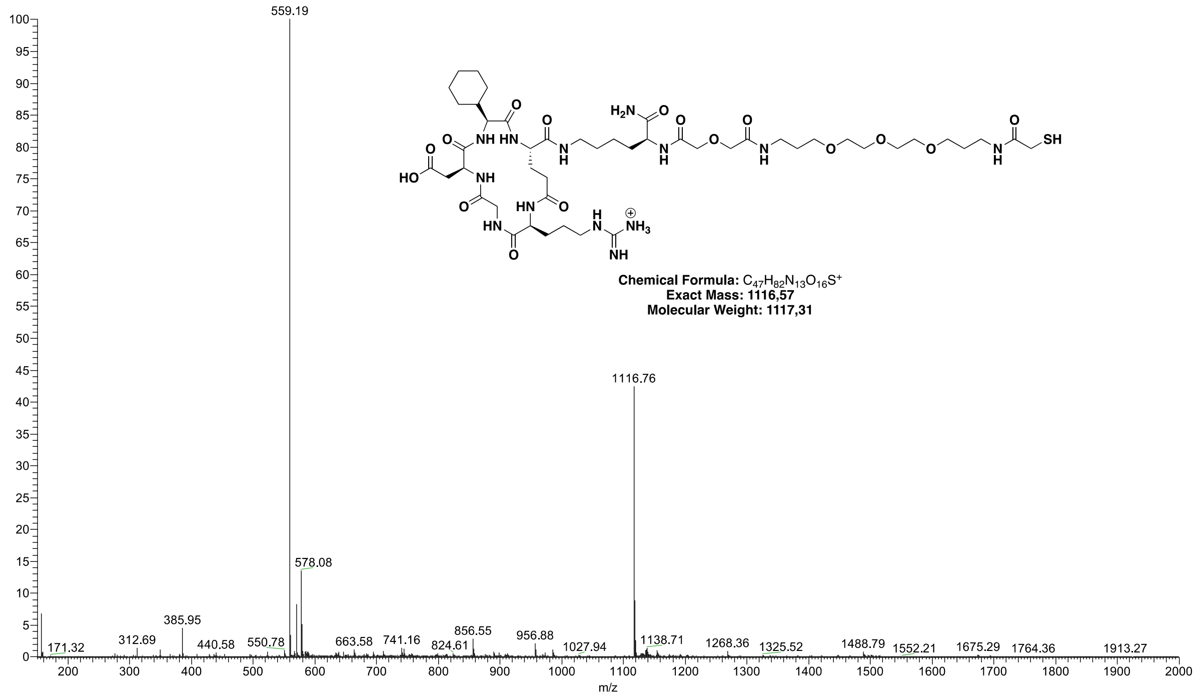

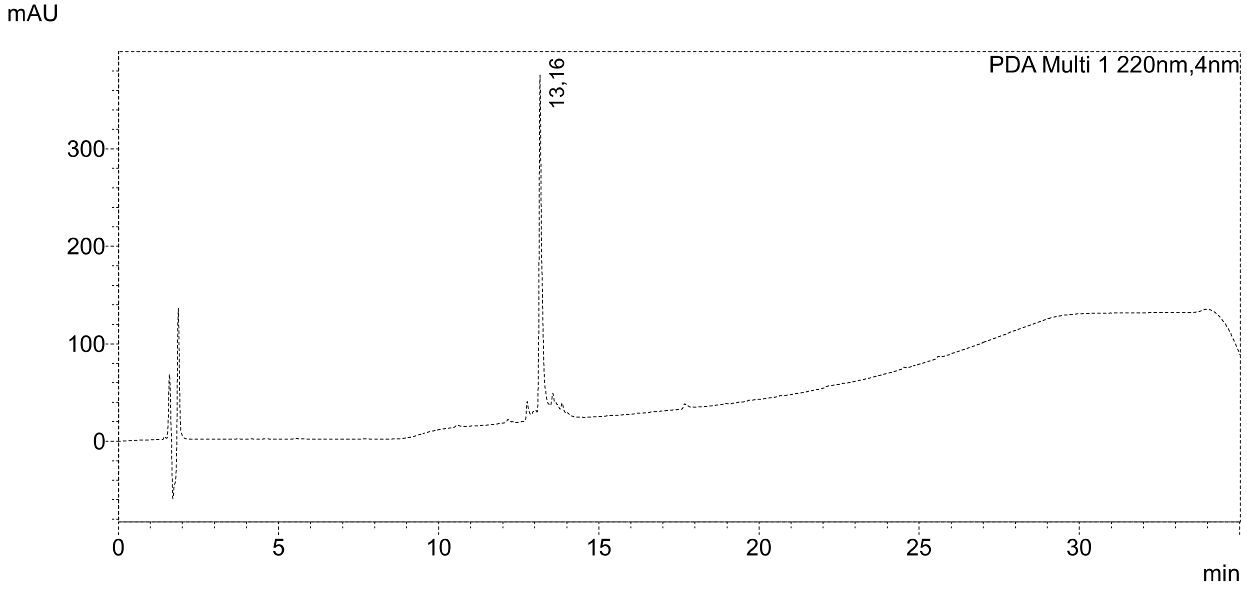

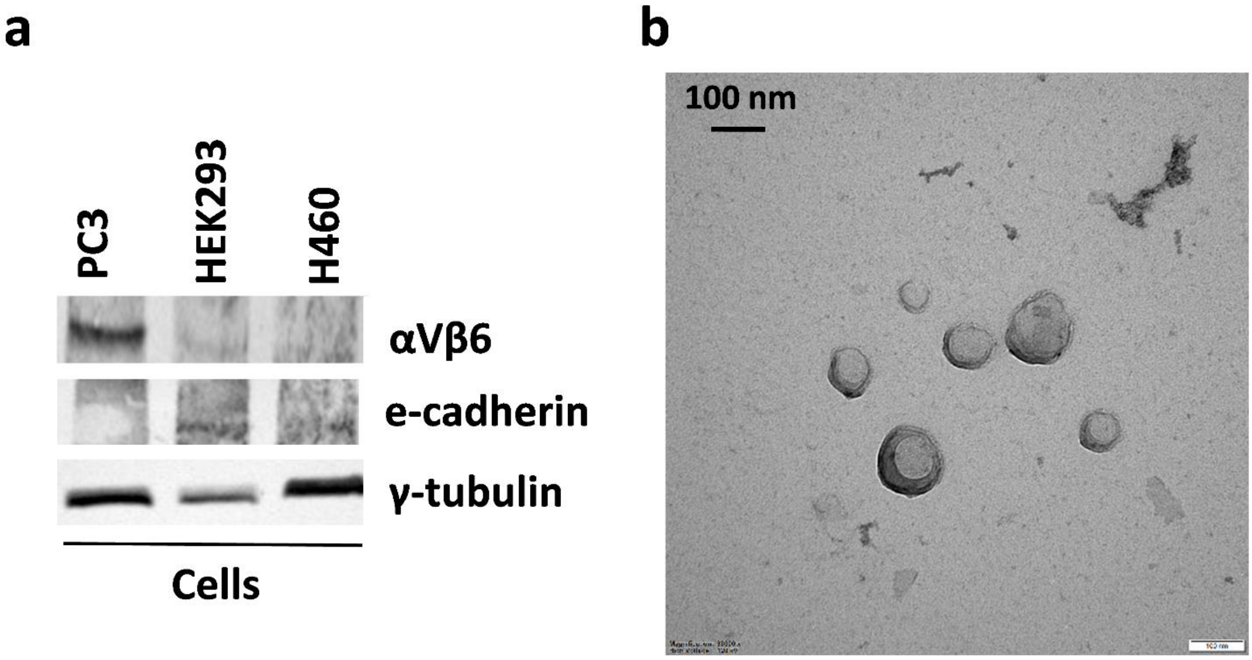

Figure S2. a) Western blot analysis of PC3, HEK293 and H460 cell lysates (25 µg) tested with antibodies as indicated in the figure, shows no expression of αVbeta6 integrin in both HEK293 and H460 in serum starved conditions. b) Representative field of IEM on S-EVs labelled with secondary antibody alone. Magnification 98000X, scale bars 100 nm.

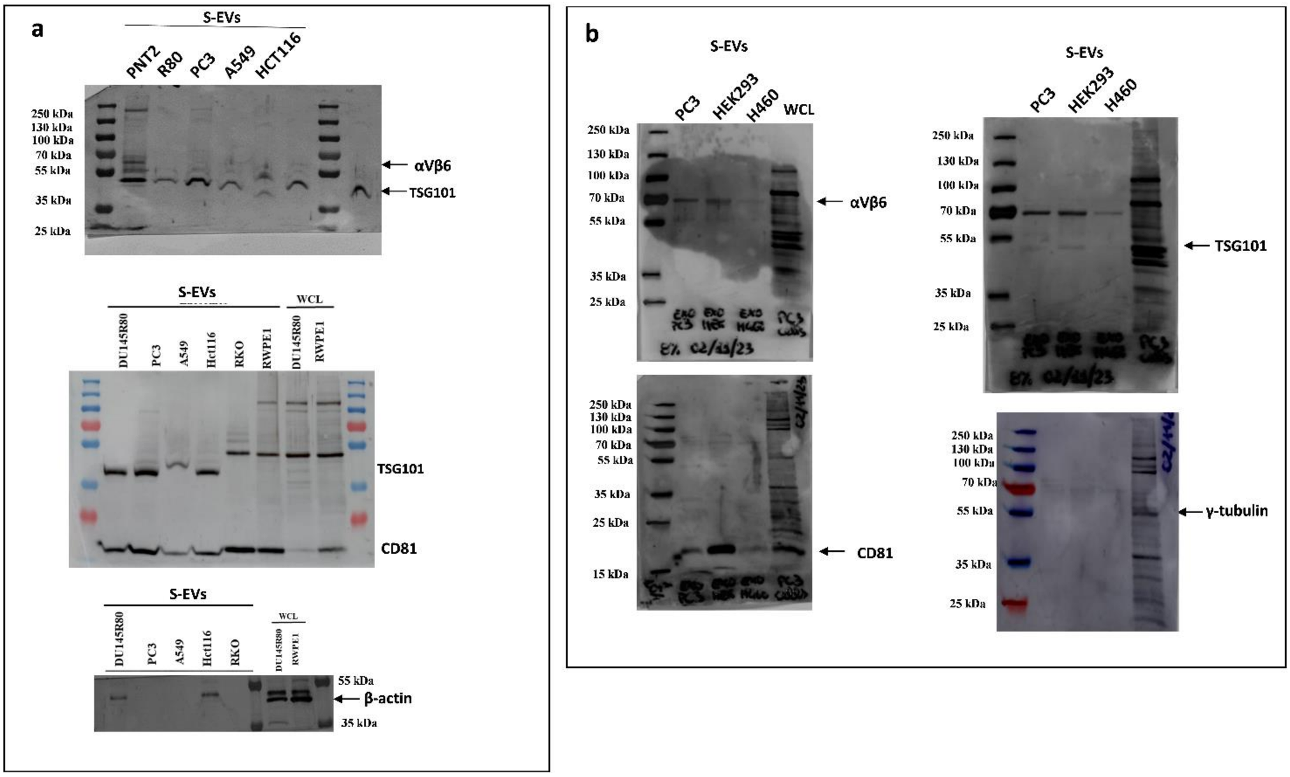

Figure S3. Uncropped blot for a) Figure 5c b) Figure 5d. WCL= whole cell lysates

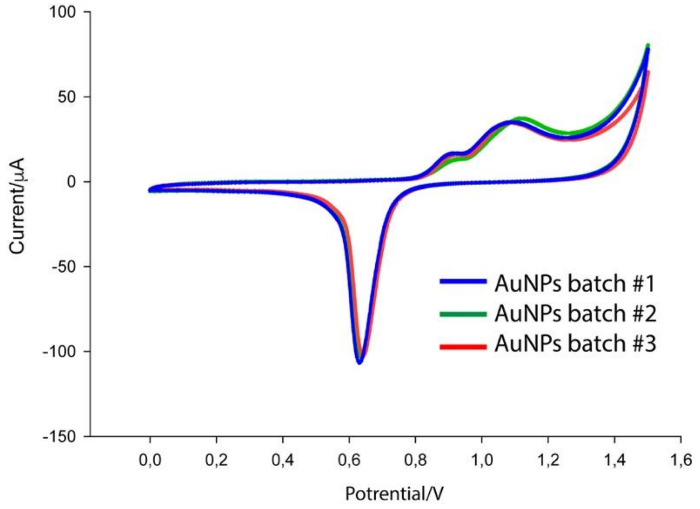

Figure S4. Cyclic voltammograms obtained in 0.1 M sulfuric acid using electrochemical strips modified with three different batches of synthesized AuNPs. Scan rate of the experiments is 0.1 V/s.

Table S2: Comparison of biosensors and biosensing approaches to determine other integrins.

Integrin Types	Technique	Detection limit	Real-sample Application	Ref
Integrin α6β4-containing cell model via IDA aptamers	DPV	14 cells/mL	A549 cells	1
Integrin β1	EIS	3.5×10^3 cells/mL	HeLa cells	2
β1 integrin	EIS	NA	MG-63 cells	3
β2-integrin	Flow Cytometry	NA	CD^{4+} and CD^{8+} T cells	4
Mouse integrin αIIbβ3	Flow cytometric	1 μg/ml	Whole Blood With JON/APE	5
αvβ3 integrin	Fluorescence	0.5 μg mL^{-1}	HT-29 cells	6
Integrins Mac-1	FRET	NA	CHO cells	7
α4-Integrin	FRET	NA	U937 cells	8
Integrin Mac-1 subunits	FRET	NA	CHO & HEK 293T cells	9
αvβ3 integrin	SERS	NA	SW620 cells	10
αvβ3 integrin	SERS	NA	Human SW620 colon cancer cells	11
αvβ3 integrin	SERS	NA	U87MG cells; MCF7 cancer cell line	12

References cited in the table:

(1) Khaksari, S.; Ameri, A. R.; Taghdisi, S. M.; Sabet, M.; Ghaani Bami, S. M. J.; Abnous, K.; Mousavi Shaegh, S. A. A Microfluidic Electrochemical Aptasensor for Highly Sensitive and Selective Detection of A549 Cells as Integrin α6β4-Containing Cell Model via IDA Aptamers. *Talanta* **2023**, *252*. https://doi.org/10.1016/j.talanta.2022.123781.

(2) Jiang, X.; Tan, L.; Zhang, B.; Zhang, Y.; Tang, H.; Xie, Q.; Yao, S. Detection of Adherent Cells Using Electrochemical Impedance Spectroscopy Based on Molecular Recognition of Integrin β1. *Sensors Actuators, B Chem* **2010**, *149*. https://doi.org/10.1016/j.snb.2010.06.026.

(3) Lin, C. Y.; Teng, N. C.; Hsieh, S. C.; Lin, Y. S.; Chang, W. J.; Hsiao, S. Y.; Huang, H. S.; Huang, H. M. Real-Time Detection of β1 Integrin Expression on MG-63 Cells Using Electrochemical Impedance Spectroscopy. *Biosens Bioelectron* **2011**, *28* (1). https://doi.org/10.1016/j.bios.2011.07.022.
(4) Schöllhorn, A.; Schuhmacher, J.; Besedovsky, L.; Fendel, R.; Jensen, A. T. R.; Stevanović, S.; Lange, T.; Rammensee, H. G.; Born, J.; Gouttefangeas, C.; Dimitrov, S. Integrin Activation Enables Sensitive Detection of Functional CD^{4+} and CD^{8+} T Cells: Application to Characterize SARS-CoV-2 Immunity. *Front Immunol* **2021**, *12*. https://doi.org/10.3389/fimmu.2021.626308.
(5) Bergmeier, W.; Schulte, V.; Brockhoff, G.; Bier, U.; Zirngibl, H.; Nieswandt, B. Flow Cytometric Detection of Activated Mouse Integrin αIIbβ3 with a Novel Monoclonal Antibody. *Cytometry* **2002**, *48* (2). https://doi.org/10.1002/cyto.10114.
(6) Shi, H.; Liu, J.; Geng, J.; Tang, B. Z.; Liu, B. Speci Fi c Detection of Integrin α. *J Am Chem Soc* **2012**, *134*, 9569–9572.
(7) Fu, G.; Wang, C.; Wang, G. ying; Chen, Y. zhang; He, C.; Xu, Z. zhan. Detection of Constitutive Homomeric Associations of the Integrins Mac-1 Subunits by Fluorescence Resonance Energy Transfer in Living Cells. *Biochem Biophys Res Commun* **2006**, *351* (4). https://doi.org/10.1016/j.bbrc.2006.10.127.
(8) Chigaev, A.; Buranda, T.; Dwyer, D. C.; Prossnitz, E. R.; Sklar, L. A. FRET Detection of Cellular A4-Integrin Conformational Activation. *Biophys J* **2003**, *85* (6). https://doi.org/10.1016/S0006-3495(03)74809-7.
(9) Fu, G.; Yang, H. yan; Wang, C.; Zhang, F.; You, Z. dong; Wang, G. ying; He, C.; Chen, Y. zhang; Xu, Z. zhan. Detection of Constitutive Heterodimerization of the Integrin Mac-1 Subunits by Fluorescence Resonance Energy Transfer in Living Cells. *Biochem Biophys Res Commun* **2006**, *346* (3). https://doi.org/10.1016/j.bbrc.2006.06.015.
(10) Sloan-Dennison, S.; Schultz, Z. D. Label-Free Plasmonic Nanostar Probes to Illuminate: In Vitro Membrane Receptor Recognition. *Chem Sci* **2019**, *10* (6). https://doi.org/10.1039/c8sc05035j.
(11) Sloan-Dennison, S.; Bevins, M. R.; Scarpitti, B. T.; Sauvé, V. K.; Schultz, Z. D. Protein Corona-Resistant SERS Tags for Live Cell Detection of Integrin Receptors. *Analyst* **2019**, *144* (18). https://doi.org/10.1039/c9an01056d.
(12) Kim, M. S.; Park, B. C.; Kim, Y. J.; Lee, J. H.; Koo, T. M.; Ko, M. J.; Kim, Y. K. Design of Magnetic-Plasmonic Nanoparticle Assemblies via Interface Engineering of Plasmonic Shells for Targeted Cancer Cell Imaging and Separation. *Small* **2020**, *16* (20). https://doi.org/10.1002/smll.202001103.
