# Supplementary material for: Paper-based electrochemical device for early detection of integrin αvβ6 expressing tumors
Source: Commun Chem. 2024 Mar 21;7:60. doi: 10.1038/s42004-024-01144-z (PMC10957923; doi:10.1038/s42004-024-01144-z)
Supplement: Supplementary file 3 — Reporting Summary [file 42004_2024_1144_MOESM3_ESM.pdf]

nature portfolio

Corresponding author(s): *Double-anonymous peer review submissions: write DAPR and your manuscript number here instead of author names.*

Last updated by author(s): *YYYY-MM-DD*

Reporting Summary

Nature Portfolio wishes to improve the reproducibility of the work that we publish. This form provides structure for consistency and transparency in reporting. For further information on Nature Portfolio policies, see our Editorial Policies and the Editorial Policy Checklist.

Statistics

For all statistical analyses, confirm that the following items are present in the figure legend, table legend, main text, or Methods section.

n/a	Confirmed	
☐	☒	The exact sample size (*n*) for each experimental group/condition, given as a discrete number and unit of measurement
☐	☒	A statement on whether measurements were taken from distinct samples or whether the same sample was measured repeatedly
☒	☐	The statistical test(s) used AND whether they are one- or two-sided<br>*Only common tests should be described solely by name; describe more complex techniques in the Methods section.*
☒	☐	A description of all covariates tested
☒	☐	A description of any assumptions or corrections, such as tests of normality and adjustment for multiple comparisons
☐	☒	A full description of the statistical parameters including central tendency (e.g. means) or other basic estimates (e.g. regression coefficient) AND variation (e.g. standard deviation) or associated estimates of uncertainty (e.g. confidence intervals)
☒	☐	For null hypothesis testing, the test statistic (e.g. *F*, *t*, *r*) with confidence intervals, effect sizes, degrees of freedom and *P* value noted<br>*Give P values as exact values whenever suitable.*
☒	☐	For Bayesian analysis, information on the choice of priors and Markov chain Monte Carlo settings
☒	☐	For hierarchical and complex designs, identification of the appropriate level for tests and full reporting of outcomes
☒	☐	Estimates of effect sizes (e.g. Cohen's *d*, Pearson's *r*), indicating how they were calculated

Our web collection on statistics for biologists contains articles on many of the points above.

Software and code

Policy information about availability of computer code

Data collection	Electrochemical experiments were performed with the software PS Trace 5.9 PalmSens
Data analysis	The electrochemical data were analyzed with Microsoft Excel 16.78.3

For manuscripts utilizing custom algorithms or software that are central to the research but not yet described in published literature, software must be made available to editors and reviewers. We strongly encourage code deposition in a community repository (e.g. GitHub). See the Nature Portfolio guidelines for submitting code & software for further information.

Data

Policy information about availability of data

All manuscripts must include a data availability statement. This statement should provide the following information, where applicable:

- Accession codes, unique identifiers, or web links for publicly available datasets
- A description of any restrictions on data availability
- For clinical datasets or third party data, please ensure that the statement adheres to our policy

The additional data supporting the findings of this study are available within the article and its Supplementary Information. The experimental datasets used to produce all the figures are available at the public repository https://zenodo.org/records/10682910.

Research involving human participants, their data, or biological material

Policy information about studies with human participants or human data. See also policy information about sex, gender (identity/presentation), and sexual orientation and race, ethnicity and racism.

Reporting on sex and gender	*Use the terms sex (biological attribute) and gender (shaped by social and cultural circumstances) carefully in order to avoid confusing both terms. Indicate if findings apply to only one sex or gender; describe whether sex and gender were considered in study design; whether sex and/or gender was determined based on self-reporting or assigned and methods used. Provide in the source data disaggregated sex and gender data, where this information has been collected, and if consent has been obtained for sharing of individual-level data; provide overall numbers in this Reporting Summary. Please state if this information has not been collected. Report sex- and gender-based analyses where performed, justify reasons for lack of sex- and gender-based analysis.*
Reporting on race, ethnicity, or other socially relevant groupings	*Please specify the socially constructed or socially relevant categorization variable(s) used in your manuscript and explain why they were used. Please note that such variables should not be used as proxies for other socially constructed/relevant variables (for example, race or ethnicity should not be used as a proxy for socioeconomic status). Provide clear definitions of the relevant terms used, how they were provided (by the participants/respondents, the researchers, or third parties), and the method(s) used to classify people into the different categories (e.g. self-report, census or administrative data, social media data, etc.) Please provide details about how you controlled for confounding variables in your analyses.*
Population characteristics	*Describe the covariate-relevant population characteristics of the human research participants (e.g. age, genotypic information, past and current diagnosis and treatment categories). If you filled out the behavioural & social sciences study design questions and have nothing to add here, write "See above."*
Recruitment	*Describe how participants were recruited. Outline any potential self-selection bias or other biases that may be present and how these are likely to impact results.*
Ethics oversight	*Identify the organization(s) that approved the study protocol.*

Note that full information on the approval of the study protocol must also be provided in the manuscript.

Field-specific reporting

Please select the one below that is the best fit for your research. If you are not sure, read the appropriate sections before making your selection.

☒ Life sciences ☐ Behavioural & social sciences ☐ Ecological, evolutionary & environmental sciences

For a reference copy of the document with all sections, see nature.com/documents/nr-reporting-summary-flat.pdf

Life sciences study design

All studies must disclose on these points even when the disclosure is negative.

Sample size	No sample size calculation
Data exclusions	No data excluded
Replication	Experiments were repeated using at least 20 tests per figures
Randomization	N/A
Blinding	N/A

Reporting for specific materials, systems and methods

We require information from authors about some types of materials, experimental systems and methods used in many studies. Here, indicate whether each material, system or method listed is relevant to your study. If you are not sure if a list item applies to your research, read the appropriate section before selecting a response.

Materials & experimental systems

n/a	Involved in the study
☐	☒ Antibodies
☐	☒ Eukaryotic cell lines
☒	☐ Palaeontology and archaeology
☒	☐ Animals and other organisms
☒	☐ Clinical data
☒	☐ Dual use research of concern
☒	☐ Plants

Methods

n/a	Involved in the study
☒	☐ ChIP-seq
☒	☐ Flow cytometry
☒	☐ MRI-based neuroimaging

Antibodies

Antibodies used

αvβ6 integrin (Bioss, bs-5791R, lot AI07297632), TSG101 (abcam cat. ab30871 lot GR3204565-1), CD81 (cell signaling #56039 lot 1), β-actin (ab 8227 lot GR3385771-1), γ-tubulin (sc-17787, lot# l3019)

Validation

αvβ6 integrin (Bioss, bs-5791R, lot AI07297632) species: Human Mouse Rat; applications: WB, ELISA, FCM, IHC-P, IHC-F, IF(IHC-P), IF(IHC-F), IF(ICC). Validations: None provided from the manufacturer. Detects a band of approximately molecular weight 75kDa
TSG101 (abcam cat. ab30871 lot GR3204565-1) species: Mouse, Rat, Human applications: WB, ICC/IF, IHC-P PMID: 35281474
Detects a band of approximately 48,49 kDa (predicted molecular weight: 44 kDa).
CD81 (D3N2D) (cell signaling #56039 lot 1) species: Human applications: WB, IHC PMID: 37357686 molecular weight: 22 kDa
β-actin (ab 8227 lot GR3385771-1) species: Mouse, Rat, Rabbit, Chicken, Cow, Dog, Human, Xenopus laevis, Fish, Chinese hamster applications: WB, IHC-P, ICC/IF Detects a band of approximately molecular weight 40kDa
γ-tubulin (C-11) (sc-17787, lot# l3019) species mouse, rat e human applications: WB, IP, IF, IHC(P) e ELISA
Detects a band of approximately molecular weight 50kDa

Eukaryotic cell lines

Policy information about cell lines and Sex and Gender in Research

Cell line source(s)

PC-3 is a cell line initiated from a bone metastasis of a grade IV prostatic adenocarcinoma from a 62-year-old, White, male and was purchased from American Type Culture Collection (Rockville, MD, USA).

DU145R80 cells have been developed from DU145 cells (see material section of the main manuscript). DU 145 is a cell line with epithelial morphology that was isolated from the brain of a 69-year-old, White, male with prostate cancer.

HCT 116 cell line was isolated from the colon of an adult male with colon cancer and purchased from American Type Culture Collection (Rockville, MD, USA).

A549 is an epithelial cell that was isolated from the lung of a 58-year-old, White male with carcinoma and was purchased from American Type Culture Collection (Rockville, MD, USA).

HEK293 is a cell line exhibiting epithelial morphology that was isolated from the kidney of a human embryo. It was purchased from American Type Culture Collection (Rockville, MD, USA).

NCI-H460 cells were isolated in 1982 from the pleural fluid of a male patient with large cell lung cancer. It was purchased from American Type Culture Collection (Rockville, MD, USA).

Authentication

All Cell lines have been profiled by LGC Standards Cell Line Authentication service by Human STR testing

Mycoplasma contamination

Cell lines regularly inspected for mycoplasma by Mycoalert Mycoplasma detection kit (Lonza), negative cells (Ratio < 0,9 following manufacturer's instructions) were used for experiments.

Commonly misidentified lines
(See ICLAC register)

Name any commonly misidentified cell lines used in the study and provide a rationale for their use.

Plants

Seed stocks	*Report on the source of all seed stocks or other plant material used. If applicable, state the seed stock centre and catalogue number. If plant specimens were collected from the field, describe the collection location, date and sampling procedures.*
Novel plant genotypes	*Describe the methods by which all novel plant genotypes were produced. This includes those generated by transgenic approaches, gene editing, chemical/radiation-based mutagenesis and hybridization. For transgenic lines, describe the transformation method, the number of independent lines analyzed and the generation upon which experiments were performed. For gene-edited lines, describe the editor used, the endogenous sequence targeted for editing, the targeting guide RNA sequence (if applicable) and how the editor was applied.*
Authentication	*Describe any authentication procedures for each seed stock used or novel genotype generated. Describe any experiments used to assess the effect of a mutation and, where applicable, how potential secondary effects (e.g. second site T-DNA insertions, mosiacism, off-target gene editing) were examined.*
